# *Sargassum fusiforme *fraction is a potent and specific inhibitor of HIV-1 fusion and reverse transcriptase

**DOI:** 10.1186/1743-422X-5-8

**Published:** 2008-01-15

**Authors:** Elena E Paskaleva, Xudong Lin, Karen Duus, James J McSharry, Jean-Claude L Veille, Carol Thornber, Yanze Liu, David Yu-Wei Lee, Mario Canki

**Affiliations:** 1 Center for Immunology and Microbial Disease, Albany Medical College, Albany, NY, USA; 2 Ordway Research Institute, Inc., Albany, NY, USA; 3 Department of Ob/Gyn, Albany Medical College, Albany, NY, USA; 4 Department of Biological Sciences, University of Rhode Island, Kingston, USA; 5 Mailman Research Center, McLean Hospital, Harvard Medical School, Belmont, MA, USA

## Abstract

*Sargassum fusiforme *(Harvey) Setchell has been shown to be a highly effective inhibitor of HIV-1 infection. To identify its mechanism of action, we performed bioactivity-guided fractionation on *Sargassum fusiforme *mixture. Here, we report isolation of a bioactive fraction SP4-2 (*S. fusiforme*), which at 8 μg/ml inhibited HIV-1 infection by 86.9%, with IC_50 _value of 3.7 μg. That represents 230-fold enhancement of antiretroviral potency as compared to the whole extract. Inhibition was mediated against both CXCR4 (X4) and CCR5 (R5) tropic HIV-1. Specifically, 10 μg/ml SP4-2 blocked HIV-1 fusion and entry by 53%. This effect was reversed by interaction of SP4-2 with sCD4, suggesting that *S. fusiforme *inhibits HIV-1 infection by blocking CD4 receptor, which also explained observed inhibition of both X4 and R5-tropic HIV-1. SP4-2 also inhibited HIV-1 replication after virus entry, by directly inhibiting HIV-1 reverse transcriptase (RT) in a dose dependent manner by up to 79%. We conclude that the SP4-2 fraction contains at least two distinct and biologically active molecules, one that inhibits HIV-1 fusion by interacting with CD4 receptor, and another that directly inhibits HIV-1 RT. We propose that *S. fusiforme *is a lead candidate for anti-HIV-1 drug development.

## Background

*S. fusiforme *is a species of brown macroalgae (Class Phaeophyceae) that is commonly found in middle to lower rocky intertidal zones along the coastlines of China, Korea, and Japan. Formerly called *Hizikia fusiformis *[1], it frequently occurs in dense aggregations. Individuals can be up to 1 m in length, with shorter side branches and narrow blades. It is frequently collected for human consumption. In our previous work with whole *S. fusiforme *extract, we reported up to 90% inhibition of HIV-1 replication in several different cell types, including T cells and macrophages, both during entry and post-entry stages of the HIV-1 life cycle [2]. Importantly, this inhibition was also mediated against primary isolate R5-tropic HIV-1 (ADA) in human macrophages, and it also inhibited cell-to-cell fusion and subsequent viral spread to uninfected cells, which demonstrated ability of *S. fusiforme *to inhibit physiologically relevant HIV-1 mechanism of infection.

Based upon this work, we proposed that *S. fusiforme *mixture contained more than one biologically active molecule, and that it would be a lead candidate for bioactivity-guided isolation of active compounds mediating HIV-1 inhibition. Here, we report the isolation of a bioactive fraction SP4-2, with 230-fold enhanced antiretroviral activity against both X4 and R5-tropic HIV-1, specificity of inhibition of viral fusion mediated against CD4 receptor, and post entry inhibition of the HIV-1 RT. Compounds isolated from *S. fusiforme *have not been investigated until now [3,4].

## Results

### Dose dependent inhibition of HIV-1

**Figure 1:**
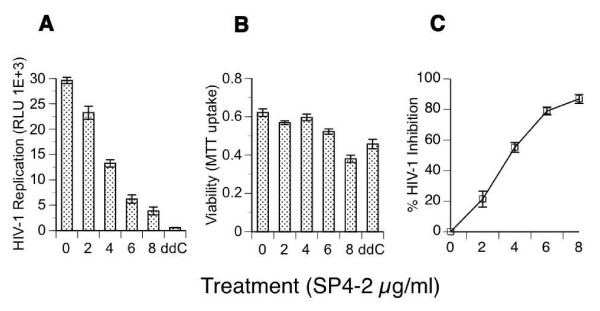
**Inhibition of HIV-1 infection**. 1G5 T cells were pretreated for 24 h with increasing concentrations of SP4-2, or with 10^-6^M ddC, or mock treated (0 μg SP4-2), as indicated. Then, cells were infected with HIV-1 (NL4-3) at multiplicity of infection (moi) of 0.01 for 1.5 h, washed 3 times, and returned to culture with the same concentration of each treatment, for the duration of the experiment. (A) On day 3 after infection, HIV-1 infection was quantified by luciferase gene marker expression from cell lysates that were normalized to the same number of viable cells, and expressed as relative light units (RLU) on the y-axis. (B) Viability for each cell culture treatment was quantified by MTT uptake. (C) Percent inhibition of HIV-1 was calculated from raw data in (A), utilizing the formula in the Methods, and plotted on the Y-axis as % HIV-1 Inhibition. Data are mean ± SD of three separate experiments.

To begin characterization of the complex S. fusiforme extract, we performed bioactivity-guided fractionation, which resulted in identification of a biologically active fraction SP4-2 that we tested in T cells for the ability to inhibit HIV-1 infection (Fig. 1). Cells were treated with increasing concentrations of SP4-2, infected, and virus replication was measured by luciferase expression in 1G5 cells that were equalized to the same number of viable cells by the MTT assay (Fig. 1A). Viability of treated cultures remained high and similar to that of mock and 10^-6^M ddC treated cells (Fig. 1B). Maximal virus replication was determined from infected and untreated cells (0 μg SP4-2), which expressed 29,601 luciferase relative light units (RLU), demonstrating active and ongoing virus replication (Fig. 1A). Highly productive infection was confirmed by flow cytometry, with 99% of cells positive for HIV-1 antigens (data not shown). Comparatively, treatment with 2 μg, 4 μg, 6 μg, and 8 μg/ml SP4-2 reduced luciferase expression in a dose-dependent manner to 23,243, 13,253, 6,222, and 3,877 RLU, respectively. As expected, control cultures treated with 10^-6^M ddC, expressed background counts of 587 RLU, indicating almost total inhibition of virus replication (Fig. 1A). We calculated percent HIV-1 inhibition in comparison to infected and untreated cells (Fig. 1C). Treatment with SP4-2 inhibited virus replication in a dose dependent manner by 21, 55, 79, and 86%, respectively. The 50% inhibitory concentration (IC_50_) was calculated to be 3.7 μg.

### *S. fusiforme *inhibits both X4 and R5-tropic HIV-1 infection

**Figure 2:**
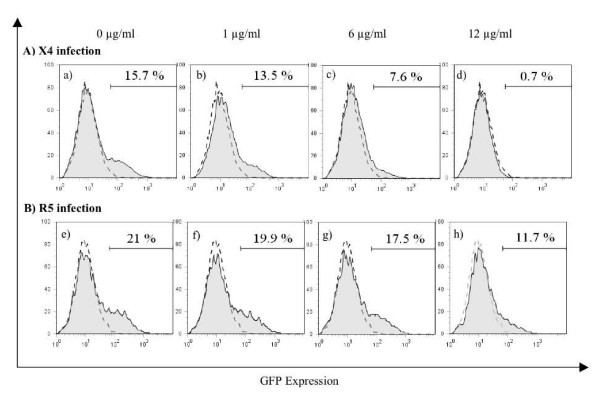
**Inhibition of X4 and R5-tropic HIV-1**. GHOST X4/R5 and GFP expressing cells were plate at 1 × 10^5^/well in 12-well plates and incubated at 37°C in CO_2 _atmosphere with increasing concentrations of SP4-2, as indicated, then infected with either X4-tropic NL4-3 (panel A, a-d) or with R5-tropic 81A (panel B, e-h), at 0.3 moi, in replicates (n = 4). 48 h after infection cells were quantified by FACS, and % infected cells is shown on each panel. Uninfected and untreated control (mock) is superimposed over each graph in dotted line. Representative of 4 experiments.

Next, we examined the cells coreceptor specificity and tested SP4-2 fraction for ability to inhibit both X4 and R5-tropic HIV-1 (Fig. 2). GHOST cells expressing both X4 and R5 coreceptors were treated with increasing concentrations of SP4-2, and infected with X4-tropic NL4-3 (A) or with R5-tropic 81A (B), and FACS analyzed 48 h after infection. Treatment with SP4-2 resulted in a dose dependent decrease in number of infected cells by either virus. X4-tropic virus (A) infected 15.7% cells without treatment (a), which decreased to 13.5% (b), 7.6% (c), and 0.7% (d) infected cells after treatment with 1, 6, and 12 μg/ml SP4-2, respectively. Inhibition of infection was calculated to be 14%, 51%, and 95%, respectively. For R5-tropic infection, we observed a mean of 21% infected cells (e), which decreased to 19.9% (f), 17.5% (g), and 11.7% (h) infected cells after treatment with 1, 6, and 12 μg/ml SP4-2, respectively. Inhibition of infection was calculated to be 6%, 17%, and 45%, respectively. However, when we increased SP4-2 treatment to 14, 16, 20, and 24 μg/ml, R5 inhibition of infection increased proportionally to 65%, 70%, 78%, and 88%, respectively (not shown). Based on these results, we conclude that treatment with SP4-2 inhibits both X4 and R5-tropic HIV-1 infection in a dose dependent manner, confirming our previous results with whole S. fusiforme extract, which inhibited both X4 and primary R5-tropic HIV-1.

### *S. fusiforme *inhibits HIV-1 fusion by blocking CD4 receptor

**Figure 3:**
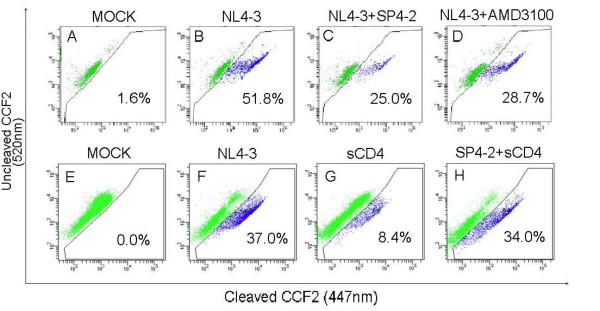
**Inhibition of HIV-1 fusion**. SupT1 cells (1 × 10^6^) were (A) mock infected, (B) infected for 2 h at 0.5 moi with BlaM-Vpr-X4-tropic NL4-3, or (C) infected in the presence of 10 μg/ml SP4-2, or (D) infected in the presence of 250 nM AMD3100. In a parallel experiment, SupT1 cells (1 × 10^6^) were either (E) mock infected, or (F) infected for 2 h at 0.5 moi with BlaM-Vpr-X4-tropic NL4-3, or (G) infected in the presence of 20 ng/ml sCD4, or (H) infected in the presence of 20 ng/ml sCD4 together with 16 μg/ml SP4-2. Cells were loaded with CCF2/AM dye and fusion was analyzed by multiparameter flow cytometry using a violet laser for excitation of CCF, and gated from 10,000 cells. Percentages in each panel are of cells displaying blue fluorescence (virus fusion positive cells). Representative of 3 separate experiments.

Viral entry into cells consists of two distinct steps of 1) virus binding to the cellular receptor and coreceptor, which is followed by 2) fusion of the viral and cellular membranes and internalization. To determine mechanism of the observed inhibition of infection, we tested for SP4-2 activity against HIV-1 fusion to CD4-expressing SupT1 T cells, by utilized a highly specific and sensitive fluorescence resonance energy transfer (FRET)-based HIV-1 fusion assay (Fig. 3), [5,6]. HIV-1 β-lactamase-Vpr (BlaM-Vpr) chimerical HIV-1 (NL4-3) was used to infect target cells that were loaded with CCF2/AM dye. Changes in CCF2 fluorescence reflect intracellular presence of BlaM, which is only present due to HIV-1 fusion and entry. Mock-treated negative control cells were loaded with dye, and were gated for background 520 nm emissions, which was low at 1.6% positive cells (0% fusion, panel A). After infection with BlaM-Vpr HIV-1, fusion was detected in 51.8% of the cells (100% fusion), as indicated by a shift to blue fluorescence (panel B). However, treatment of cells with 10 μg SP4-2 fraction inhibited this shift and markedly reduced viral entry, with only 25% of the cells being positive for viral fusion, which corresponded to 51.7% inhibition of the fusion (panel C). As a positive control for inhibition, we treated cells with 250 nM AMD3100 (CXCR4 inhibitor), which inhibited virus fusion, yielding 28.7% fusion positive cells that corresponded to 44.5% inhibition (panel D). Inhibition of fusion with AMD3100 increased to 80%, when we increased its concentration to 500 nM (not shown). From three different experiments we observed that treatment with 10 μg SP4-2 inhibited HIV-1 fusion by average of 53% (± 0.8 SEM).

Next, in a parallel experiment, we studied for the possible interaction between SP4-2 and CD4 (Fig. 3E–H). From 37% BlaM-Vpr HIV-1 fusion positive cells without any inhibitor (panel F), incubation with sCD4 only, resulted in 8.4% positive cells and blocked HIV-1 fusion by 77.2% (panel G). However, incubation of sCD4 together with SP4-2 resulted in 34% HIV-1 fusion positive cells (panel H), in effect reversing inhibition of fusion observed with sCD4 treatment. This result clearly indicates that SP4-2 interacts with CD4 receptor thereby blocking HIV-1 fusion to target cell.

### *S. fusiforme *inhibits HIV-1 binding but not entry or replication

**Figure 4:**
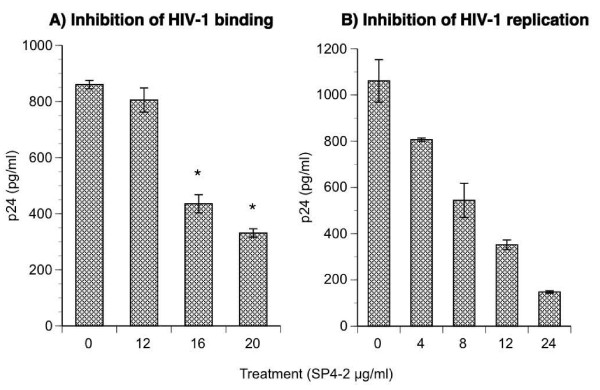
**Inhibition of HIV-1 binding and replication**. GHOST cells were plate at 1 × 10^5^/well in 12-well plates and incubated at 37°C in CO_2 _atmosphere with increasing concentrations of SP4-2 for 1.5 hours prior to infection. Treatment was washed off 3 times with warm media and plates were transferred to 4°C for 2 h to cool. Then the cells were infected at 4°C with NL4-3 at 0.1 moi for 2 hours. (A) Unbound virus was removed by washing with cold PBS, and viral particles remaining bound to the cells were quantified by p24 ELISA. (B) In a parallel experiment, 4°C infected plates were returned to 37°C for 48 hours, and virus replication was quantified by p24 ELISA. Data are mean ± SD of 6 replicates.

In addition to demonstrating inhibition of HIV-1 fusion by SP4-2-CD4 interaction, we were interested to define mechanism of this inhibition by investigating whether treatment with S. fusiforme prevents virus binding to the cell surface receptors in culture (Fig. 4). Cells that are infected at 4°C allow only HIV-1 binding to the cell surface receptor but not fusion or entry. Except for 2 h SP4-2 pretreatment of cells that was done at 37°C to allow for SP4-2-CD4 interaction, we performed all the subsequent steps, including HIV-1 infection at 4°C. GHOST X4/R5 expressing cells were treated with increasing concentrations of SP4-2 (0–20 μg), and then washed three times with warm media to remove any unbound SP4-2. Next, cells were cooled and infected at 4°C with NL4-3 for 2 h, washed three times to remove any unbound virus, and bound HIV-1 was quantified from replicates (n = 6) by HIV-1 core antigen p24 ELISA (Fig. 4A). Treatment with 0, 12, 16, and 20 μg/ml SP4-2, resulted in a dose dependent decrease of HIV-1 bound to cells, which measured 860, 805, 435, and 331 pg/ml p24, respectively. The percent decrease in bound virus was calculated comparative to 100% bound virus (860 pg/ml p24), which was 6.3, 49.4, and 61.5%, respectively. Treatment with both 16 and 20 μg SP4-2 led to statistically significant decrease (p ≤ 0.0001) compared to no treatment (0 μg). To test whether HIV-1 bound at 4°C was capable of membrane fusion and replication, in a parallel experiment performed under same conditions, we returned the infected and washed cell cultures to 37°C for 48 h, and quantified virus replication by monitoring HIV-1 p24 production (Fig. 4B). Cell cultures pretreated with 0, 4, 8, 12, and 24 μg/ml SP4-2, replicated HIV-1 in a dose dependent manner that produced 1061, 807, 544, 352, and 148 p24 pg/ml, respectively. The HIV-1 inhibition was calculated to be 23.9, 48.7, 66.8, and 86%.

### *S. fusiforme *inhibits HIV-1 reverse transcriptase

**Figure 5:**
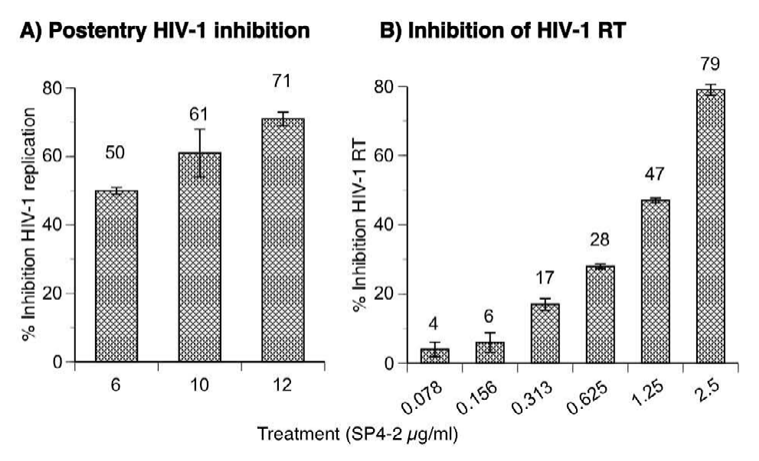
**Inhibition of post entry HIV-1 replication**. (A) SupT1 cells were infected for 1.5 hours in the absence of any treatment, with HIV-1 chimera NL4-3 Env^-^Luc^+^/VSV-G pseudotype, washed 3 times, and then treated with increasing concentrations of SP4-2, for 24 h. Intracellular luciferase gene marker expression was quantified from cell lysates that were normalized to the same number of viable cells by the MTT assay, and percent inhibition of HIV-1 replication was calculated from a control cell culture of infected but untreated cells, and plotted on the y-axis. (B) Standard cell free fluorescent RT assay was performed in the presence of 2 units recombinant HIV-1 RT/reaction with the indicated concentrations of SP4-2. Percent inhibition was calculated comparative to assay performed in absence of treatment, 100% RT activity. Data are mean ± SD of three separate experiments.

We showed that inhibition by whole S. fusiforme was mediated during several stages of the virus life cycle [2]. To determine mechanism of this inhibition, we examined HIV-1 replication during post entry steps of the virus replication cycle (Fig. 5). HIV-1 that is envelope deficient and is pseudotyped with VSV-G envelope bypasses any receptor entry restrictions and allows for a single round of infection, as previously demonstrated [7]. To bypass inhibition at entry, we infected SupT1 cells with NL4-3 Env^-^Luc^+ ^virus pseudotyped with VSV-G envelope for 2 h, and then added increasing concentrations of SP4-2 treatment. 24 h after infection, we measured luciferase production and calculated inhibition of virus replication in response to SP4-2 treatment (Fig. 5A). Treatment with 6, 10, and 12 μg SP4-2 inhibited post entry HIV-1 replication in a dose dependent manner by 50, 61, and 71%, respectively. Viability of treated cells, as quantified by MTT assay, remained similar to mock treatment (data not shown).

These data demonstrate that the HIV-1 is inhibited by SP4-2 after virus entry into cells. To examine the precise mechanism of the observed post entry inhibition, we investigated direct inhibition of recombinant HIV-1 RT, in a cell free assay. Treatment with increasing concentrations of SP4-2, with 0.078, 0.156, 0.313, 0.625, 0.125, and 2.5 μg, inhibited HIV-1 RT activity in a dose dependent manner by 4, 6, 17, 28, 47, and 79%, respectively (Fig. 5B). As a negative control for inhibition, we used a different fraction that was derived from whole S. fusiforme, which was shown to be inactive during bioactivity-guided fractionation. This fraction did not inhibit HIV-1 RT (not shown).

## Discussion

Recently, we identified whole *S. fusiforme *extract as a potent inhibitor of HIV-1 infection, which at a concentration of 3 mg/ml lowered viral infection by up to 80% in a variety of primary cells and cell lines, and for a prolonged period of time [2]. To begin identification of the active components that are contained within this extract, we started bioactivity-guided fractionation that resulted in identification of a biologically active fraction SP4-2, which at 8 μg/ml inhibited HIV-1 infection by 86.9% (Fig. 1). Compared with the IC_50 _value of 860 μg to the whole extract previously reported by us, SP4-2 inhibited virus replication with an IC_50 _value of 3.7 μg, which represents a 230-fold enrichment of the antiretroviral activity. Importantly, SP4-2 treatment did not decrease cell viability, which remained similar to either mock or ddC treated controls (Fig. 1B). Interestingly, SP4-2 inhibited both X4 and R5-tropic HIV-1 infections in a dose dependent manner (Fig. 2). Although SP4-2 was more potent in inhibiting X4 virus (compare Fig. 2A to 2B), when we increased SP4-2 dose, we observed corresponding dose dependent increase in R5 virus inhibition of up to 88%, without lowering cell viability (data not shown). The observed differences in inhibition of infection can be explained due to innate differential expression of coreceptors on GHOST cells. However, inhibition of both X4 and R5 HIV-1, suggested no specificity for inhibition of HIV-1 coreceptors. To ascertain mechanistic specificity of inhibition observed by bioactive SP4-2 fraction, we next performed detailed analysis of HIV-1 fusion events (Fig. 3). Indeed, in three separate experiments, treatment with 10 μg SP4-2 inhibited HIV-1 fusion by an average of 53% (Fig. 3C). As a positive control for inhibition of fusion, both AMD3100 and sCD4 also inhibited HIV-1 entry, as expected (Fig. 3D and 3G, respectively). We further examined specificity of this inhibition, by investigating whether SP4-2 might reverse the observed sCD4 inhibition of HIV-1 fusion, and we tested this possibility by preincubating SP4-2 together with sCD4 (Fig. 3H). Indeed, SP4-2 almost completely reversed sCD4 inhibition of HIV-1 fusion, presumably by binding to it. Inhibition of CD4 receptor also explains observed dual inhibition of both X4 and R5-tropic HIV-1 infection (Fig. 2), since both strains utilize CD4 as their main receptor.

To further clarify these events, we examined ability of SP4-2 fraction to directly inhibit HIV-1 binding to cellular surface receptors in culture (Fig. 4). HIV-1 infection at 4°C allows only binding of the virus to cellular receptors but not membrane fusion or cellular entry. Cells treated with increasing concentrations of SP4-2 and infected at 4°C, inhibited HIV-1 binding in a dose dependent manner by up to 61% (Fig. 4A). Next, to test whether 4°C bound HIV-1 was able to fuse, enter cells and replicate, in a parallel experiment, we returned 4°C infected cultures to 37°C for 48 h and measured HIV-1 replication by p24 ELISA (Fig. 4B). Similar to inhibition of HIV-1 binding, SP4-2 also inhibited virus replication in a dose dependent manner. This result confirmed our data for inhibition of fusion (Fig. 3), demonstrating that *S. fusiforme *blocks HIV-1 entry by interfering with virus binding to CD4 receptor on cell surface.

Whole *S. fusiforme *extract inhibited cell-to-cell fusion and viral spread to the uninfected cells, however it also inhibited post fusion events of HIV-1 replication life cycle [2]. To investigate mechanism of post entry inhibition, we tested ability of the SP4-2 fraction to inhibit HIV-1 replication after bypassing entry restriction (Fig. 5). We first infected cells with NL4-3 Env^-^Luc^+^/VSV-G that bypasses any receptor restrictions and allows for one round of virus replication [7]. After completing the infection, cells were treated with increasing concentrations of SP4-2, which inhibited virus replication in a dose dependent manner by up to 71%, clearly demonstrating post entry inhibition of viral life cycle (Fig. 5A).

First step after HIV-1 entry is reverse transcription and cDNA formation by viral RT, and therefore we next investigated for possible direct inhibition of HIV-1 RT by SP4-2, in a cell free assay (Fig. 4B). Indeed, SP4-2 inhibited HIV-1 RT in a dose dependent manner by up to 79%. Importantly, as a negative control, we also tested a similar fraction that was derived from whole *S. fusiforme *extract, which did not have any biological activity, including RT inhibition (not shown).

To examine specificity of *S. fusiforme *inhibition of HIV-1, we also tested for possible inhibition of two additional enveloped viruses, vaccinia and influenza, which were not inhibited by SP4-2 (data not shown). Unlike nonspecific inhibition by sulfated polysaccharides isolated from natural sources [8-10], *S. fusiforme *does not inhibit infection of the enveloped viruses that we tested. Instead, its specificity of inhibition for HIV-1 can be explained through its particular interaction with the viral CD4 receptor and direct inhibition of reverse transcriptase.

## Conclusion

Taken together, we have demonstrated an average of 53% inhibition of HIV-1 fusion, and approximately 47% of virions that do enter cells are further inhibited up to 79% by RT, which equals to a total global inhibition of HIV-1 infection of approximately 90% that is in agreement with our results (Fig. 1). These results show that the SP4-2 fraction contains two distinct inhibitory activities against HIV-1, which we hypothesize to be mediated by at least two different molecules, one that is CD4 fusion inhibitor and the other that is RT inhibitor. We conclude that *S. fusiforme *is a lead candidate for HIV-1 antiviral drug development.

## Materials and methods

### Bioactivity-guided fractionation

A sample of *S. fusiforme *(14 kg) was soaked in aqueous 70% acetone (140 L × 2) overnight. The filtered extract was concentrated to remove the acetone and the residue was dried overnight. The extraction temperature was controlled at 70°C to avoid possible thermal breakdown of bioactive natural products. The solid residue was filtered to give 75 g of a dark blue paste (SP4), with activity similar to that of the whole aqueous extract generated previously [2]. SP4 (38 g) was dissolved in 200 ml of methanol and treated with 10 g of active charcoal. After filtration, the brown solution was concentrated, yielding 14 g of brown residue, which was subjected to silica gel column chromatography and eluted with methylene chloride with an increasing amount of methanol. A total of 600 fractions (25 ml/each) were collected and grouped into 27 fractions following TLC analyses. The SP4-2 (fraction #81–120, 903 mg) was the most active fraction in 1G5 luciferase assay monitoring inhibition of HIV-1. Further purification of SP4-2 to its individual components is currently in progress.

### Cells

1G5 [11], SupT1 [12], and GHOST X4/R5 [13] cells were obtained from the HIV AIDS Research and Reference Reagent Program, Division of AIDS, NIAID, NIH, and were cultured and maintained as specified by the reagent protocol. Cells were treated as indicated in the Figure legends for each experiment, infected at the indicated moi, washed three times, and returned to culture with the indicated concentration of each treatment, for the duration of experiment, and then analyzed as indicated.

### HIV-1 molecular clones, envelope expression vectors, and generation of pseudotyped and BlaM-Vpr chimera

HIV-1 X4-tropic molecular clone NL4-3 expresses all known HIV-1 proteins [14], and the R5-tropic molecular clone 81A-4 has Ba-L Env sequences on the backbone of NL4-3 [15] were obtained from HIV AIDS Research and Reference Reagent Program. Envelope expression deficient and luciferase positive pNL4-3.HSA.R^+^.E^- ^was obtained from Dr. Nathaniel Landau [16,17], and was pseudotyped with VSV-G envelope to produce single round infectious HIV-1. pL-VSV-G vector was obtained from Dr. M. Emerman; it contains a VSV G insert in the pcDNA expression vector modified by replacing the cytomegalovirus promoter with the HIV-1 long terminal repeat [18]. We generated native and pseudotyped virus as previously described [7]. Briefly, 1.5 × 10^6 ^293T cells cultured in 10-cm^2 ^plates were cotransfected by calcium phosphate precipitation [19], with 10 μg of HIV-1 clone DNA and 15 μg of VSV-G envelope expression plasmid DNA, a ratio of DNAs found to yield the highest HIV-1 infectious titers in our hands. For native HIV-1 production, 1.5 × 10^6 ^293T cells were transfected with 15 μg of NL4-3 or 81A DNA. 293T culture supernatants were harvested 72 h after transfection, filtered through a 0.45-μm-pore-size Millipore filter, and stored at -80°C until use. Cell-free viral stock was quantified for HIV-1 p24 core antigen content by enzyme-linked immunosorbent assay (ELISA) using the HIV-1 Ag kit as specified by the manufacturer (AIDS Vaccine Program, NCI-Frederick), and was also quantified for titers of infectious virus by multinuclear activation of a β-galactosidase indicator (MAGI) assay [20]. Culture supernatants contained 1 to 2 μg of viral p24 protein per ml and 1 × 10^6 ^to 2 × 10^6 ^infectious units (IU) per ml. In our hands, a multiplicity of infection of 1 for CD4-positive T cells is equivalent to approximately 1 pg of viral p24 per cell [7].

Fusion sensitive BlaM-Vpr chimera DNA plasmid was a kind gift from Dr. W. Greene [5], and HIV-1 virions containing the BlaM-Vpr chimera were produced as previously described [5] Briefly, 293T cells in 10 cm^2 ^flasks were cotransfected with pNL4-3 proviral DNA (60 μg), pCMV-BlaM-Vpr (20 μg), and pAdVAntage vectors (10 μg) (Invitrogen). After 48 h at 37°C, the virus-containing supernatant was centrifuged at low speed to remove cellular debris and at 72,000 *g *for 90 min at 4°C to concentrate virus, which was resuspended in DMEM and aliquoted for storage at -80°C. For all transfections, calcium phosphate was used to precipitate DNA, and viral stocks were normalized by p24 content measured by ELISA as described above.

### Infection and analysis of HIV-1 expression by luminescence, FACS, and RT

For determination of luciferase expression, 1G5 T cells were seeded in 12 well plates at 1 × 10^6 ^cells/well, treated for 24 h as indicated in Figure legend, then washed to remove treatment, and infected in replicates at the indicated moi. After washing, cells were returned to culture with the same concentration of each treatment for 3 days, and then equal number of viable cells that were normalized by a CellTiter 96 Non-Radioactive Cell Proliferation Assay [(3-(4,5-Dimethyl-2-thiazolyl)-2,5-dephenyltetrazolium, Promega] (MTT) assay, were tested for luciferase expression using a Luciferase Assay System (Promega), as specified by the manufacturer.

Percent (%) inhibition was determined utilizing the following formula:

$$Inhibition[\%]=\left[1-\frac{\text{(T}reatedcells\text{)}-\text{(}Mock-treatedcells\text{)}}{\text{(}Untreatedcells\text{)}-\text{(}Mock-treatedcells\text{)}}\right]\times 100$$

Fusion assay was done as previously described [5,6]. Briefly, Sup T1 cells were first infected for 2 h with BlaM-Vpr-X4 (NL4-3) chimera at 0.5 moi, washed in CO_2 _independent media and loaded for 1 h at room temperature (rt) with the CCF2/AM dye as specified by the manufacturer (Gibco), washed in developing buffer and reaction was allowed to developed overnight. After development, cells were washed in PBS and fixed in 1.2% paraformaldehyde solution. BlaM reaction was detected by the change in emission fluorescence of CCF2 after cleavage by the BlaM-Vpr chimera, which was monitored by FACS with a three-laser Vantage SE (Becton Dickinson, San Jose, CA). A coherent krypton laser operating at 200 mW and generating light at 406.7 nm was used to excite the CCF2 dye. Blue emission was detected with an HQ455/50 filter, and green emission was detected with an HQ545/90 BP filter; for light splitting, a 505 SP filter was used. Data were collected with CellQuest and analyzed with FlowJo software (Treestar, San Carlos, CA).

GHOST X4/R5 expressing adherent cells that are stably transfected with GFP under control of the HIV-1 LTR, and cells were plated in 24-well plates at concentration of 5 × 10^4 ^cells/well in 90% DMEM, 10% fetal bovine serum, 500 mg/ml G418, 100 mg/ml hygromycin, 1 mg/ml puromycin, and 1% penicillin/streptomycin. Next day cells were treated with 2-fold dilutions of 50 mg/ml SP4-2 for 1.5 hours. The treatment was then removed by washing, and cells were infected at 0.3 moi with either X4-tropic (NL4-3), or with R5-tropic (81A) HIV-1 clone. Infection was carried out in a volume of 150 μl at 37°C in 5% CO_2 _atmosphere, cell cultures were washed and returned to media containing each respective treatment. Cells were collected 40–48 hours post infection, washed in PBS, and incubated in 200 μl 1.2% parafolmaldehyde in PBS for 2–3 hours at 4°C prior to FACS analysis. Cell counting was performed on BD FACSCanto™ FACS system and analyzed with BD FACSDiva software. The percent of infected (GFP-expressing) cells in untreated wells was taken as 100% infection and inhibition by SP4-2 was calculated comparative to it.

HIV-1 reverse transcriptase (RT) assay kit (Invitrogen) was performed in accordance with the manufacturer's instructions. Briefly, 2 units of HIV-1 RT (Ambion) were mixed in the reaction mixture with the indicated serial dilutions of SP4-2, and RT activity was quantified from fluorescence readings resulting from RT catalyzing RNA-DNA heteroduplex formation. Percent RT inhibition was calculated from RT reaction in the absence of treatment or 100% RT activity.

## Competing interests

The author(s) declare that they have no competing interests.

## Authors' contributions

MC, EEP, and DYWL participated in the design of experiments.

MC, EEP, XL, and DYWL participated in the interpretation of the results.

MC and EEP prepared the manuscript.

EEP, XL, KD, JJM, JCV, CT, and YL performed the experiments.
